# Context-Based Development to Promote Physical Activity Among Working-Age Populations: Participatory Action Research and Pilot Test

**DOI:** 10.3390/ijerph23010087

**Published:** 2026-01-08

**Authors:** Kamlai Somrak, Poramet Hemarachatanon, Saranrat Manunyanon, Kiattisak Pechpan, Phiphat Khlongdi, Sanhapan Wattanapisit, Apichat Photia, Apichai Wattanapisit

**Affiliations:** 1 Department of Community Nursing, School of Nursing, Walailak University, Nakhon Si Thammarat 80160, Thailand; 2 Department of Health Promotion, Walailak University Hospital, Nakhon Si Thammarat 80160, Thailand; 3 The Excellent Center of Community Health Promotion, Walailak University, Nakhon Si Thammarat 80160, Thailand; 4 Department of Sport and Exercise Science, School of Medicine, Walailak University, Nakhon Si Thammarat 80160, Thailand; 5 Center for Cultural and Sports Promotion, Walailak University, Nakhon Si Thammarat 80160, Thailand; 6 Department of Social Medicine, Thasala Hospital, Nakhon Si Thammarat 80160, Thailand; 7 Division of Clinical and Metabolic Genetics, The Hospital for Sick Children, Toronto, ON M5G 1E8, Canada; 8 Department of Clinical Medicine, School of Medicine, Walailak University, Nakhon Si Thammarat 80160, Thailand; 9 Family Medicine Clinic, Walailak University Hospital, Nakhon Si Thammarat 80160, Thailand

**Keywords:** health promotion, physical activity, Thailand, working-age population

## Abstract

Insufficient physical activity (PA) is a significant health challenge among working-age populations. This study aimed to develop context-specific processes to promote PA among adults aged 35–60 years. A participatory action research approach was conducted across seven provinces in upper southern Thailand. The study consisted of three phases: (1) preparation and situation analysis, (2) development and implementation of PA promotion programs, and (3) program evaluation and lessons learned. In Phase 1, the working-age population was categorized into four groups: Group 1: PA occupation and exercise; Group 2: PA occupation but non-exercise; Group 3: non-PA occupation but exercise; Group 4: non-PA occupation and non-exercise. In Phase 2, an exercise program and PA tracking guide were developed and implemented over a 6-month period. In Phase 3, based on the complete-case analysis, 175 participants enrolled, with 101 (57.7%) and 100 (57.1%) remaining at 3 and 6 months, respectively. Based on the last observation carried forward analysis (*n* = 175 across the 6 months), the proportion achieving global recommended PA levels and time spent in weekly moderate- to vigorous-intensity PA increased significantly in the non-exercise groups (Groups 2 and 4). All participants in the exercise groups (Groups 1 and 3) met the recommended PA level at baseline; however, this level was not maintained at the endpoint. The context-based PA promotion programs improved PA participation among non-exercise working-age adults. Future research should identify strategies to enhance program uptake and sustain engagement.

## 1. Introduction

Insufficient physical activity (PA) is a global public health challenge. Approximately one-third of adults (31.3%) engage in insufficient PA [1]. Prevalence has steadily increased over the past two decades [1]. Insufficient PA is a major contributor to adverse health outcomes, particularly non-communicable diseases (NCDs) [2,3]. Although one study reported improvements between 2012 and 2019, insufficient PA remains a significant national concern in Thailand [4]. The prevalence among Thai adults further increased during the coronavirus disease 2019 pandemic [5]. A recent survey indicated that 31.1% of adults in Thailand had insufficient PA [6]. Moreover, a prevalence of around 30% or higher has been documented in specific groups, such as university students [7,8].

Compared with the general population, the Thai working-age group has a lower prevalence of insufficient PA, at 22.3% [6]. In this population, PA may occur during leisure time or through work-related activities, such as workplace exercise or occupational tasks [9]. The benefits of PA extend beyond health outcomes and physical fitness; it can also enhance job performance, improve wellbeing, and reduce sickness-related absenteeism [9,10]. These findings highlight the importance of promoting PA among working-age adults. Nevertheless, multiple barriers hinder engagement in PA, including job characteristics, socioeconomic factors, financial limitations, time constraints, and personal behaviors [11,12,13].

A recent systematic review reported positive effects of most PA-led workplace health interventions, including increased PA levels and reduced psychological stress [14]. However, a cluster randomized trial conducted in Thailand found no significant difference in PA levels between office workers in the intervention and control groups [15]. Workplace-based interventions may not be applicable to all occupations and work settings. For example, fishermen and farmers do not work in office environments; therefore, interventions for these groups may require shifting the focus from the workplace to the broader community. Community-wide campaigns may be more feasible and adaptable to the diverse characteristics of working-age populations [16]. An illustrative example is a study conducted among Irish farmers, which examined the impact of a community-based physical activity and health education intervention [17]. This program included one health education session and two circuit exercise sessions per week over six weeks [17].

The mixed effects of previous interventions and the diversity of work characteristics highlight important gaps in knowledge and practice regarding PA promotion in working-age populations. In particular, promoting PA across varied cultural and sociodemographic contexts requires an understanding of real-world settings and potential implementation challenges. This study aimed to develop context-specific processes to promote PA among working-age populations in southern Thailand.

## 2. Materials and Methods

### 2.1. Study Design and Context

This participatory action research was conducted in the Health Region 11, which covers seven provinces in upper southern Thailand: Chumphon, Krabi, Nakhon Si Thammarat, Phang Nga, Phuket, Ranong, and Surat Thani. Each province has several subdistrict health-promoting hospitals (community health centers), which are responsible for primary health care in the public sector. Fourteen community health centers were selected as convenience sampling to participate in this study based on the researchers’ community health network. The study sites included the catchment areas of the seven provinces as well as Walailak University, located in Nakhon Si Thammarat (one of the seven provinces). The study was conducted between July 2024 and October 2025. This article was prepared in accordance with best practices for reporting participatory action research, as outlined by Smith et al. [18].

### 2.2. Target Populations

The research team consisted of multidisciplinary members, including a primary care physician, a community nurse, sports scientists, a project coordinator, and a project manager. At least 14 health professionals—such as public health officers and nurses—as well as village health volunteers from the 14 community health centers were involved. In addition, up to 20 working-age adults living in the catchment areas of the study sites were a target population for a pilot test for implementing the PA promotion program. The inclusion criteria were 35–60 years old, no known diagnosis of major NCDs (i.e., cardiovascular diseases, cancers, chronic respiratory diseases, and diabetes), and living in the catchment areas during the study. Participants would be excluded if they were pregnant, had limitations to PA participation (e.g., joint pain, personal health concerns), or could not understand the Thai language.

### 2.3. Developmental Processes, Data Collection, and Data Analysis

#### 2.3.1. Phase 1: Preparation and Situation Analysis

The research team conducted brainstorming sessions to identify PA behaviors among the working-age population in the study sites. Based on the characteristics of this population, occupational PA and leisure-time PA (i.e., exercise) were used to categorize participants into four groups: (i) PA occupation and exercise, (ii) PA occupation but non-exercise, (iii) non-PA occupation but exercise, and (iv) non-PA occupation and non-exercise. PA occupation was defined as one that involved moderate-intensity PA with an energy expenditure of ≥3.0 metabolic equivalents for most of the working time [19]. Exercise was defined according to current aerobic PA recommendations, that is, engaging in ≥150 min per week of moderate- to vigorous-intensity PA (MVPA) [20]. In this context, exercise was further characterized as “PA that is planned, structured, repetitive, and purposive in the sense that the improvement or maintenance of one or more components of physical fitness is an objective” [21].

The research team organized a workshop on developing context-based PA promotion for working-age populations in July 2024, at Walailak University. Participants included the research team and representatives from each community health center, such as health professionals and village health volunteers. The workshop comprised knowledge sharing, experiential learning activities (e.g., basic physical fitness tests, body composition measurement), the design of context-specific PA promotion plans, and group brainstorming and discussion to identify gaps and opportunities for implementing context-based PA promotion programs. The research team used the Global Physical Activity Questionnaire (GPAQ) to collect PA data from the workshop participants as a self-reported questionnaire which was commonly used in Thailand [22,23].

A topic on the categorization of the four groups was discussed among the research team based on the data obtained from the GPAQ and feedback from the workshop participants. Based on the feedback, the GPAQ would not be an easy tool to collect PA data during the program implementation. In addition, other objective measurement tools, such as accelerometers and smartwatches, were not available for the use for all participants in different study sites. The research team would use a self-reported data to interpret the PA occupation based on PA intensity during major work activities using the 2024 Adult Compendium of Physical Activities [19], and meeting PA recommendation by a shorter questionnaire, the Exercise Vital Sign [24,25,26,27].

#### 2.3.2. Phase 2: Development and Implementation of Physical Activity Promotion Programs

The research team visited the target sites to collaborate with healthcare providers and community members in preparing for the implementation of PA promotion programs. Feedback and needs were explored through discussions with representatives from each site.

The research team reviewed the outputs obtained from Phase 1 and the site visits to design implementation plans and develop communication materials for the working-age population. An exercise demonstration video and four PA tracking guide booklets were produced. Circuit exercise programs were designed by a sports scientist in our team as an optional guidance for healthcare providers and participants. PA promotion programs were not strict to the circuit exercise programs; however, they were flexible based on the contexts of diverse communities among study sites.

Subsequently, the research team visited each target site and organized a community forum to encourage participation and provide education on PA, including exercise demonstrations. In each area, two healthcare providers or village health volunteers were assigned to monitor and follow up with participants. Up to 20 working-age adults, categorized into four groups (five participants per group: (i) PA occupation and exercise, (ii) PA occupation but non-exercise, (iii) non-PA occupation but exercise, and (iv) non-PA occupation and non-exercise), were purposively selected in each area to a pilot study.

Each participant received a PA tracking guide booklet. Three outcomes were assessed: body composition, health-related physical fitness, and exercise participation. Body composition—including body weight (kg), body mass index (kg/m^2^), body fat percentage (%), muscle mass percentage (%), visceral fat (levels 1–9), and resting metabolic rate (kcal)—was measured using bioelectrical impedance analysis (Tanita model BC-730; Tanita, Tokyo, Japan). Health-related physical fitness was evaluated with three tests: (i) the sit-and-reach test for flexibility, (ii) the 60-s chair stand test for muscle strength and endurance, and (iii) the 3-min step test for cardiovascular endurance. Before the health-related physical fitness tests, participants were encouraged to warm up by instructed dynamic stretching and brief and light aerobic dance. The research team or trained staff conducted the physical fitness tests. Criteria to classify each test as “pass” or “fail”, stratified by age and sex, were based on the Thailand Department of Physical Education’s manual [28]. Exercise participation was assessed using the Exercise Vital Sign, which consists of two questions: (i) “On average, how many days per week do you engage in moderate to strenuous PA or exercise?” and (ii) “On average, how many minutes per day do you engage in moderate to strenuous PA or exercise?” [24,25,26]. Engaging in at least 150 min per week of MVPA was considered sufficient PA [20]. Outcome measurements were conducted at baseline, 3 months, and 6 months.

During the 6-month program, the in-charge healthcare providers or village health volunteers could contact the project coordinator if they had any questions or encountered problems related to the PA promotion program. The research team regularly communicated with healthcare providers at the study sites via group and personal chats on a messaging application.

#### 2.3.3. Phase 3: Program Evaluation and Lessons Learned

The research team evaluated the participatory action research processes using multiple methods, including discussions, written feedback, statistical analyses, and qualitative and quantitative data collected from healthcare providers and working-age participants, as well as quantitative outcome measurements.

At 3 months, the research team visited each target site to measure outcomes and conducted four focus group discussions, organized by participant group. The discussion topics included: (i) PA behavior before participating in the program, (ii) viewpoints on the program (e.g., participant grouping and selection, program activities), (iii) factors associated with behavior and outcome changes, and (iv) recommendations for program improvement. In each group, one participant who demonstrated favorable outcomes and improvements was selected as a role model and was invited to share key successes, outcome changes, and positive experiences.

At 6 months, participants, in-charge healthcare providers, and village health volunteers from all sites were invited to a two-day, one-night event conducted in Krabi province. The research team organized a public hearing to obtain feedback from participants and community representatives. In addition, activities were conducted to empower participants and encourage sustainable PA promotion. Finally, the research team collaborated with participants and community representatives to summarize lessons learned and develop future action plans using the Ottawa Charter for Health Promotion as a framework [29].

For statistical analyses, R version 4.0.2 (RStudio, Boston, MA, USA) was used. Categorical variables were presented as frequencies and percentages. Continuous variables were tested for normality using the Kolmogorov–Smirnov test. Normally distributed variables were reported as means and standard deviations, whereas non-normally distributed variables were reported as medians and interquartile ranges. Analytic statistics were considered significant when *p* < 0.05.

Baseline characteristics—including sex, age, PA participation, body composition, and physical fitness—were described for participants who completed outcome measurements at baseline (month 0), follow-up (month 3), and endpoint (month 6). Differences in characteristics were analyzed using Fisher’s exact test or the Chi-square test for categorical variables, and the ANOVA F-test (parametric) or Kruskal–Wallis test (non-parametric) for continuous variables.

Analyses of PA participation, body composition, and physical fitness were conducted using two approaches: a complete-case analysis and an analysis incorporating imputed data for missing outcomes. Comparisons of outcomes at baseline (month 0), follow-up (month 3), and endpoint (month 6) were performed using Fisher’s exact test or the Chi-square test for categorical variables, and the ANOVA F-test (parametric) or Kruskal–Wallis test (non-parametric) for continuous variables.

The complete-case analysis included only participants who had available outcome measurements at all relevant time points, allowing an examination of program uptake among completers and program feasibility. To address potential bias from participant dropout, an additional analysis was conducted using the last observation carried forward (LOCF) method, in which missing follow-up or endpoint values were replaced with the most recent available measurement (e.g., values at the baseline or follow-up). For the LOCF analysis, effect sizes were and 95% confidence intervals calculated to quantify the precision and magnitude of within-group changes, comparing outcomes between endpoint and baseline. Effect sizes were calculated for categorical variables using risk ratios (RR) or risk differences (RD) when the denominator was zero, and for continuous variables using Cohen’s d for normally distributed variables or Cliff’s delta for non-normally distributed variables.

## 3. Results

### 3.1. Phase 1: Preparation and Situation Analysis

The research team initially categorized the working-age population into four groups. This framework was used to guide the planning and design of the intervention.

A total of 56 participants (51 females; 91.1%) from seven provinces in upper southern Thailand attended a workshop on developing context-based PA promotion for working-age populations in July 2024. Table 1 summarizes the identified needs and recommendations for designing the context-based PA promotion program.

### 3.2. Phase 2: Development and Implementation of Physical Activity Promotion Programs

The research team designed 6-month circuit exercise programs for each group (PA occupation and exercise; PA occupation but non-exercise; non-PA occupation but exercise; non-PA occupation and non-exercise) as prototypes that incorporated aerobic PA, strengthening PA, and multicomponent PA to meet the recommended PA levels. A 31-min video demonstrating the exercise programs was provided. However, these exercise programs were adaptable based on participants’ readiness, health conditions, and preferences. Table 2 summarizes the exercise programs for each group.

**Figure 1:**
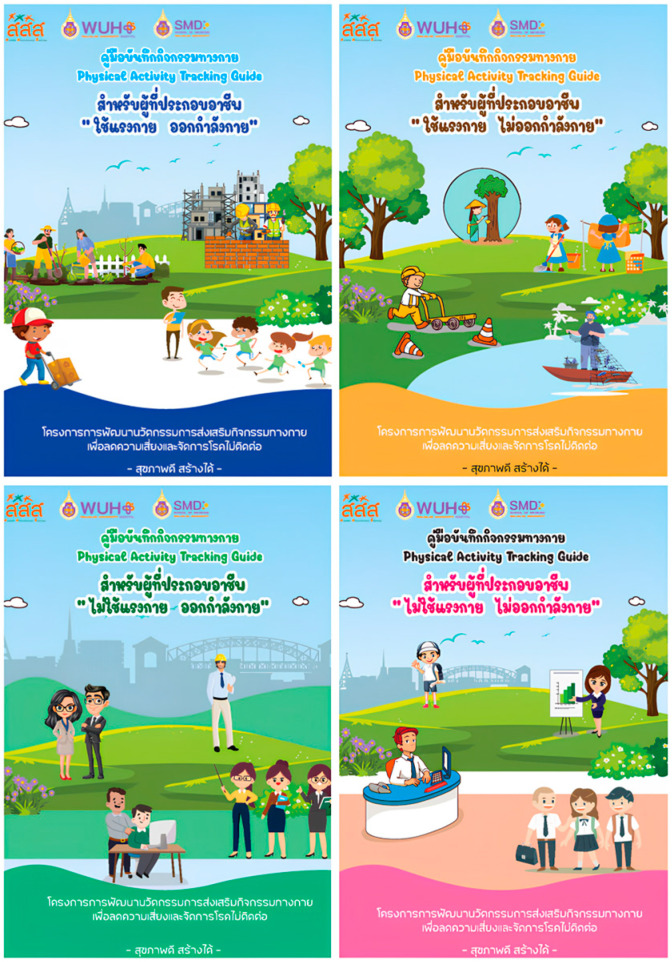
Physical activity tracking guides for participants (PA occupation and exercise (blue), PA occupation but non-exercise (orange), non-PA occupation but exercise (green), non-PA occupation and non-exercise (pink)).

Four PA tracking guides were developed, consisting of (i) basic knowledge of PA, exercise, and sedentary behavior; (ii) an exercise program; (iii) pictorial exercise illustrations; and (iv) exercise and body weight tracking tools (Figure 1).

The context-based PA promotion programs for working-age populations were implemented in seven provinces in upper southern Thailand, covering the catchment areas of 14 community health centers. A total of 175 participants were recruited at month 0 (156 females; 89.1%) with a mean age of 50.2 ± 5.9 years. Participants were classified into four groups: Group 1: PA occupation and exercise (*n* = 45; 25.7%), Group 2: PA occupation but non-exercise (*n* = 48; 27.4%), Group 3: non-PA occupation but exercise (*n* = 38; 21.7%), and Group 4: non-PA occupation and non-exercise (*n* = 44; 25.1%). The median MVPA was 120.0 min/week (interquartile range: 30.0, 210.0).

### 3.3. Phase 3: Program Evaluation and Lessons Learned

#### 3.3.1. Outcome Measurements and Program Evaluation

A total of 175 participants completed the outcome measurements at baseline, while 101 (57.7%) and 100 (57.1%) participants remained at follow-up and endpoint, respectively. Baseline characteristics of completers acorss the study period were not statistically significant (Table 3). Based on the complete-case analysis, weekly MVPA increased significantly in the non-exercise groups (Group 2: PA occupation but non-exercise and Group 4: non-PA occupation and non-exercise). The percentage of muscle mass increased significantly in all groups (Supplementary Table S1).

The LOCF analysis included all 175 participants, and all significant comparisons between baseline and endpoint are shown in Table 4. Overall, muscle mass percentage improved significantly, with mean values of 30.94% at baseline and 35.17% at endpoint (Cliff’s delta −0.25, small effect size). The proportion of participants passing the muscle strength and endurance test increased significantly from 0.83 to 0.94 (risk ratio 1.12). Changes in the proportion of participants meeting the aerobic PA recommendation were observed across all groups. An increasing trend was found in the non-exercise groups—Group 2 (PA occupation but non-exercise) and Group 4 (non-PA occupation and non-exercise)—whereas a decreasing trend was observed in the exercise groups—Group 1 (PA occupation and exercise) and Group 3 (non-PA occupation but exercise). Time spent in MVPA increased significantly in Group 2 (PA occupation but non-exercise), with a large effect size, and in Group 4 (non-PA occupation and non-exercise), with a moderate effect size. The complete analyses are presented in Supplementary Table S2.

#### 3.3.2. Lesson Learned

Lessons learned and future action plans were summarized according to the actions of the Ottawa Charter for Health Promotion (Table 5).

## 4. Discussion

This study illustrated the processes involved in developing context-based PA promotion programs for working-age populations. The program was introduced as an adaptable guide that could be modified according to local needs and participant characteristics. Out of the expected 280 participants (20 participants from each of the 14 community health centers), 175 enrolled in the 6-month program, and 100 (57.1%) remained at the endpoint (month 6). PA participation increased significantly among non-exercise groups, indicating the program’s potential to engage individuals with previously low activity levels.

In this study, working-age populations were categorized based on PA in occupation and exercise. These categories were informed by PA domains, which include work, travel, and recreation or leisure [30,31]. A study by Fujioka (2025) reported that work-related PA was the largest contributor to MVPA among adults in the United States, accounting for 50.6%, followed by recreational PA at 39.0% [30]. Together, these two domains represented approximately 90% of total MVPA in US adults [30]. In contrast, in Japan, active travel contributed 42.9% of MVPA, while recreational and work-related PA accounted for 30.8% and 27.5%, respectively [30]. These findings illustrate notable differences in the contribution of travel-related PA between countries, although work and recreational PA remain major MVPA sources in adult lives [30]. In our study, the travel domain was excluded due to its variability across populations and settings. For the recreational or leisure domain, we focused specifically on “exercise”, as this term is more commonly understood among the Thai population than the broader term “physical activity” [32].

The guided exercise programs in this study were based on circuit training. However, most participants did not use the exercise booklets or follow the circuit training routines as intended. This reflected communication and implementation challenges among the research team, community health centers, and participants. Circuit training was selected because of its demonstrated effectiveness in improving body composition and physical fitness, and its capacity to incorporate multiple types of PA such as aerobic and strengthening exercises [33,34,35]. Although 6-month circuit training has the potential to improve health outcomes, this type of exercise is more suitable for settings that can organize participants to attend supervised training sessions, such as university-based settings [34]. In our study, participants were more inclined to engage in other feasible and familiar activities, such as community-organized aerobic dance or neighborhood walking. To provide flexible and evidence-based options, future programs may consider offering a variety of PA strategies to increase participation, such as “weekend warrior” patterns (accumulating recommended PA within 1–2 days per week) and approaches to reduce sedentary behavior during work hours, including light-intensity PA and “exercise snacking,” which involves brief bouts of exercise integrated into the daily routine [36,37].

This study aimed to recruit 280 participants from 14 community health centers across seven provinces; however, 175 individuals (62.5%) enrolled in the program. Notably, a number of older adults in the catchment areas expressed interest and participated in the PA promotion activities. This was a favorable outcome for community health centers, as it demonstrated their capacity to proactively promote PA within the community. At the same time, only 57.1% of enrolled participants (100/175), or 37.5% of the expected sample (100/280), remained at the end of the program. These findings reflect the challenges of both reaching and retaining working-age populations in PA promotion efforts. In addition to addressing the high attrition rate, we analyzed the baseline characteristics of completers at baseline, follow-up, and endpoint, which did not differ significantly. A LOCF analysis was conducted using data from all 175 participants. Apart from statistical analyses, this study used a participatory action research approach to develop prototypes of context-based PA promotion programs. Future research should focus on examining the implementability, scalability, and sustainability of these programs in broader settings [38]. The RE-AIM framework (Reach, Effectiveness, Adoption, Implementation, and Maintenance) may serve as a useful model for guiding and evaluating future implementation efforts [39].

The proportions of participants meeting the aerobic PA recommendations differed significantly among the groups. However, two distinct interpretive directions were observed. In the exercise groups (Groups 1 and 3), all participants met the aerobic PA recommendations at baseline. The proportions declined over the 6-month follow-up period. This suggests that the programs were unable to maintain aerobic PA among participants who were already active. In contrast, among the non-exercise groups (Groups 2 and 4), the percentages of meeting the aerobic PA recommendations increased across the follow-up periods. This trend was more evident in Group 2 (PA occupation)—from 0% to 21% to 35%—compared with Group 4 (non-PA occupation), which increased from 0% to 2% to 11%. This finding aligns with previous research showing that individuals with higher levels of occupational activity are more likely to meet the recommended PA levels [40]. However, in our study, self-reported MVPA focused on exercise. Participants in the PA occupation but non-exercise group were more likely to increase their aerobic PA than their counterparts in the non-PA occupation and non-exercise group. This contrasts with earlier evidence suggesting that individuals with active occupations are less likely to increase exercise participation than those with sedentary jobs [41].

The focus of this study was the development of PA promotion programs for working-age populations. The programs did not rely solely on exercise training interventions; rather, they comprised a comprehensive process to promote PA based on the context of each study site. Working-age populations are not a primary target for PA research in Thailand [22]. Among the few studies focusing on this population, a randomized controlled trial conducted in the Thai context reported no significant differences in PA levels between office workers in the intervention and control groups [15]. The intervention in that trial was developed based on the social-ecological model, addressing individual, social, environmental, and organizational levels [15]. In contrast, our study employed a participatory action research approach, collaborating with community health centers across diverse community-based contexts. A community-based PA intervention conducted among Irish farmers shared several similarities with our study, including community-based settings, circuit exercise training, and pre- and post-intervention assessments [17]. That intervention demonstrated significant improvements in body composition and physical fitness over a six-week period [17]. Key factors that may have contributed to these outcomes included a clearly structured schedule comprising 12 exercise and workshop sessions over six weeks [17]. Although the intervention was implemented in a smaller group (*n* = 30) and over a shorter duration compared with our study, participant attrition remained a challenge [17].

We extracted lessons learned according to the health promotion action areas outlined in the Ottawa Charter for Health Promotion [29]. We found that several PA promotion activities remained highly dependent on the research team. Therefore, the core strategies, including advocacy, enablement, and mediation [29], should guide communication and collaboration between the research team and community health centers. For long-term sustainability, empowering the community is essential [42]. Accordingly, capacity building is needed to enable community partners to maintain the programs independently. To reduce dropouts and improve adherence to PA promotion programs, several strategies, such as incentives, reminders, and phone or digital follow-ups, may be applied [43].

This study had several limitations. First, the project was conducted across diverse real-world contexts, and the development of PA promotion programs was adapted to each setting, which resulted in variations in program design and implementation. Second, many community health centers depended on support from the research team for outcome measurements. Participants who were unable to attend activities during site visits may not have had their outcomes assessed. Third, standardized outcome measurements using bioelectrical impedance analysis were diverse across the sites and events due to logistic and management factors. For example, the research team could not control the time of day and fasting status for the measurements. Fourth, PA participation and time spent in MVPA were collected subjectively using questionnaires. This may lead to social desirability biases. In addition, the research team did not test the reliability of the Thai version of the Exercise Vital Sign questions. However, the English version had excellent reliability (intraclass correlation coefficient 0.98, *p* < 0.01) compared to the accelerometer [25]. Fifth, some healthcare providers responsible for program delivery were retired or transferred during the study period, which affected continuity of program implementation. Lastly, the predominance of female participants may limit the generalizability of the findings, particularly to male working-age populations.

## 5. Conclusions

This study demonstrated the development of context-based PA promotion programs from preparation and situation analysis through to program implementation and evaluation. The context-based PA promotion programs may have the potential to improve PA participation among non-exercise working-age adults. Program uptake and long-term sustainability, however, remain key challenges. In particular, challenges such as limited use of standardized exercise routines and resource constraints highlight the need for flexible, context-sensitive approaches and ongoing support in real-world implementation. Future research should focus on strategies to improve participant engagement and support the sustained implementation of context-based PA promotion in community settings.

## Figures and Tables

**Table 1:** Needs and recommendations for the context-based PA promotion program.

Main Topic	Detail
Knowledge	1.1 Appropriate exercise types for the age group and occupational demands 1.2 Safe and correct exercise techniques and self-care practices 1.3 Knowledge of stretching exercises and basic fitness assessment
Skills	2.1 Exercise skills, including stretching and strengthening exercises 2.2 Project management skills (e.g., recruiting target participants and organizing activities) 2.3 Conducting basic physical fitness assessments 2.4 Measuring PA participation
Support	3.1 Equipment and budget for body composition assessments 3.2 Knowledge transfer to local leaders in the catchment area 3.3 Community-based activities (e.g., contests, project scale-up)
Team participation	4.1 Site visits and mentoring provided by the core research team 4.2 Promoting sustainable exercise activities within the community 4.3 Context-based program adaptation

**Table 2:** Exercise programs.

Group	Detail
Group 1: PA occupation and exercise	Frequency: 2 days/week Intensity: moderate Time: 60 min/session Type: − Warm up: dynamic stretching 10–15 min − Circuit training 30 min (1 set) − Cool down: static stretching 10–15 min Progress: increase the circuit training from 1 to 3 sets and the frequency from 2 to 3 days/week in 4th to 6th month
Group 2: PA occupation but non-exercise	Frequency: 3 days/week Intensity: moderate Time: 60 min/session Type: − Warm up: dynamic stretching 10–15 min − Circuit training 30 min (1 set) − Cool down: static stretching 10–15 min Progress: increase the circuit training from 1 to 2 sets in 4th to 6th month
Group 3: Non-PA occupation but exercise	Frequency: 3 days/week Intensity: moderate Time: 60 min/session Type: − Warm up: dynamic stretching 10–15 min − Circuit training 30 min (1 set) − Cool down: static stretching 10–15 min Progress: increase the circuit training from 1 to 2 sets in 4th to 6th month Sedentary break advice messages
Group 4: Non-PA occupation and non-exercise	Frequency: 3 days/week Intensity: moderate Time: 60 min/session Type: − Warm up: dynamic stretching 10–15 min − Circuit training 30 min (2 set) − Cool down: static stretching 10–15 min Progress: increase the number of reps/set from 12–20 to 20 reps in 4th to 6th month Sedentary break advice messages

**Table 3:** Baseline characteristics of completers.

Baseline Characteristic of Completer	Baseline (Month 0) *n* = 175	Follow-Up (Month 3) *n* = 101	Endpoint (Month 6) *n* = 100	*p*-Value
Sex				
Female (n, (%)) Male (n, (%))	156 (89.1) 19 (10.9)	90 (89.1) 11 (10.9)	85 (85.0) 15 (15.0)	0.552 ^a^
Age (median, (IQR)) [years]	51.0 (47.0, 54.5)	51.0 (47.0, 55.0)	51.0 (47.0, 56.0)	0.919 ^b^
PA participation				
Passing aerobic recommendation (n, (%))	83 (47.4)	46 (45.5)	45 (45.0)	0.914 ^a^
Passing muscle-strengthening recommendation (n, (%))	64 (36.6)	36 (35.6)	38 (38.0)	0.941 ^a^
Passing multicomponent (n, (%))	56 (32.0)	33 (32.7)	31 (31.0)	0.968 ^a^
MVPA (median, (IQR)) [min/week]	120.0 (30.0, 210.0)	120 (40.0, 225.0)	102.5 (30.0, 202.5)	0.962 ^b^
Body composition				
Body weight (mean ± SD) [kg]	63.5 ± 12.3	62.1 ± 11.9	63.8 ± 12.2	0.550 ^c^
Body mass index (median, (IQR)) [kg/m^2^]	24.3 (22.4, 27.3)	24.0 (22.0, 27.0)	24.4 (22.5, 27.3)	0.700 ^b^
Body fat percentage (median, (IQR)) [%]	34.1 (31.0, 37.9)	33.8 (30.8, 38.0)	33.5 (30.3, 37.8)	0.795 ^b^
Muscle mass percentage (median, (IQR)) [%]	27.9 (23.4, 37.3)	33.8 (24.1, 37.5)	34.3 (24.2, 39.1)	0.204 ^b^
Visceral fat (median, (IQR)) [levels 1–9]	7.0 (5.5, 10.0)	7.0 (5.5, 9.0)	7.5 (5.5, 10.0)	0.487 ^b^
Resting metabolic rate (mean ± SD) [kcal]	1280.0 ± 193.0	1240.0 ± 182.0	1280.0 ± 191.0	0.241 ^c^
Physical fitness tests (passing scores)				
Flexibility (n, (%))	140 (85.5)	88 (87.1)	84 (84.0)	0.819 ^a^
Muscle strength and endurance (n, (%))	145 (83.3)	86 (85.1)	80 (80.0)	0.613 ^a^
Cardiovascular endurance (n, (%))	77 (44.3)	48 (47.5)	43 (43.0)	0.796 ^a^

^a^ Chi-square test; ^b^ Kruskal–Wallis test; ^c^ ANOVA F-test. IQR: interquartile range, MVPA: moderate- to vigorous-intensity physical activity, PA: physical activity, SD: standard deviation. Normally distributed continuous variables are presented in means and standard deviations. Non-normally distributed continuous variables are presented in medians and interquartile ranges.

**Table 4:** Significant outcomes based on the last observation carried forward analysis (n = 175).

Significant Outcome by Group	Baseline (Month 0)	Endpoint (Month 6)	Effect Sizes and 95%CI
All groups (*n*= 175)			
Muscle mass percentage (mean, (95%CI)) [%]	30.94 (29.64 to 32.24)	35.17 (33.74 to 36.59)	−0.25 (−0.36 to −0.13) ^c^
Passing muscle strength and endurance test (proportion, (95%CI))	0.83 (0.77 to 0.88)	0.94 (0.89 to 0.96)	1.12 (1.04 to 1.21) ^a^
PA occupation and exercise (Group 1) (*n* = 45)			
Meeting aerobic recommendation (proportion, (95%CI))	1.00 (0.92 to 1.00)	0.89 (0.76 to 0.95)	0.89 (0.80 to 0.98) ^a^
Muscle mass percentage (mean, (95%CI)) [%]	32.60 (30.01 to 35.19)	37.52 (34.63 to 40.41)	−0.29 (−0.50 to −0.05) ^c^
PA occupation but non-exercise (Group 2) (*n* = 48)			
Meeting aerobic recommendation (proportion, (95%CI))	0 (0 to 0.07)	0.35 (0.23 to 0.50)	0.35 (0.22 to 0.49) ^b^
Meeting muscle-strengthening recommendation (proportion, (95%CI))	0.10 (0.04 to 0.22)	0.33 (0.22 to 0.47)	3.2 (1.27 to 8.04) ^a^
Meeting multicomponent recommendation (proportion, (95%CI))	0 (0 to 0.07)	0.27 (0.16 to 0.40)	0.27 (0.14 to 0.40) ^b^
Time spent in MVPA (mean, (95%CI)) [min/week]	50.00 (37.91 to 62.09)	124.79 (89.45 to 160.14)	−0.48 (−0.65 to −0.26) ^c^
Muscle mass percentage (mean, (95%CI)) [%]	31.16 (28.76 to 33.56)	35.68 (33.26 to 38.09)	−0.30 (−0.49 to −0.07) ^c^
Passing muscle strength and endurance test (proportion, (95%CI))	0.79 (0.65 to 0.88)	0.96 (0.86 to 0.99)	1.22 (1.04 to 1.43) ^a^
Non-PA occupation but exercise (Group 3) (*n* = 38)			
Meeting aerobic recommendation (proportion, (95%CI))	1.00 (0.91 to 1.00)	0.82 (0.66 to 0.91)	0.82 (0.70 to 0.95) ^a^
Muscle mass percentage (mean, (95%CI)) [%]	30.30 (27.10 to 33.50)	33.89 (30.89 to 36.89)	−0.22 (−0.46 to −0.04) ^c^
Non-PA occupation and non-exercise (Group 4) (*n* = 44)			
Meeting aerobic recommendation (proportion, (95%CI))	0 (0 to 0.08)	0.11 (0.05 to 0.24)	0.11 (0.02 to 0.21) ^b^
Time spent in MVPA (mean, (95%CI)) [min/week]	36.36 (25.64 to 47.09)	65.68 (49.31 to 82.05)	−0.64 (−1.08 to −0.21) ^d^

^a^ risk ratio; ^b^ risk difference; ^c^ Cliff’s delta; ^d^ Cohen’s d. 95%CI: 95% confidence interval, MVPA: moderate- to vigorous-intensity physical activity, PA: physical activity. Group 1: PA occupation and exercise (≥3.0 METs for most of the working time and ≥150 min/week of MVPA from exercise). Group 2: PA occupation but non-exercise (≥3.0 METs for most of the working time and <150 min/week of MVPA from exercise). Group 3: Non-PA occupation but exercise (<3.0 METs for most of the working time and ≥150 min/week of MVPA from exercise). Group 4: Non-PA occupation and non-exercise (<3.0 METs for most of the working time and <150 min/week of MVPA from exercise).

**Table 5:** Summary of lessons learned and action plans.

Component	Lessons Learned	Action Plan
Building healthy public policy	Policies supporting clear guidelines and standardized procedures for intervention delivery and outcome measurement are needed.	Develop and disseminate a standardized implementation guideline for program facilitators.
Creating a supportive environment for health	Limited physical environments in the community were a barrier to PA participation and restricted available PA options.	Collaborate with community stakeholders to expand opportunities for PA participation and compensate for limited physical environments.
Strengthening community action	Community health centers played a key role in connecting with community members and facilitating community-based actions.	Collaborate with community health centers to support and promote context-based PA activities.
Developing personal skills	Exercise skills for participants and coaching skills for healthcare providers were needed to enhance stakeholders’ confidence in PA participation and support.	Provide capacity-building activities and online consultations to strengthen essential exercise and coaching skills among healthcare providers.
Reorienting health services	Health promotion programs, including PA promotion, were feasible to integrate into community health center services; however, monitoring and outcome measurement should be incorporated into routine practice.	Support community health centers to integrate PA promotion, monitoring, and outcome measurement into their routine services.

## Data Availability

The original contributions presented in this study are included in the article/Supplementary Materials. Further inquiries can be directed to the corresponding author.

## Supplementary Materials

The following supporting information can be downloaded at: https://www.mdpi.com/article/10.3390/ijerph23010087/s1, Table S1: Outcome measurements (complete-case analysis). Table S2. Outcome measurements (last observation carried forward).

## Abbreviations

LOCF	Last observation carried forward
MET	Metabolic equivalent
MVPA	Moderate- to vigorous-intensity physical activity
PA	Physical activity
