# Supplementary material for: Context-Based Development to Promote Physical Activity Among Working-Age Populations: Participatory Action Research and Pilot Test
Source: Int J Environ Res Public Health. 2026 Jan 8;23(1):87. doi: 10.3390/ijerph23010087 (PMC12841169; doi:10.3390/ijerph23010087)
Supplement: Supplementary file 1 [file ijerph-23-00087-s001.zip › ijerph-4015546-supplementary.pdf]

Supplementary Table S1. Outcome measurements (complete-case analysis).

Outcome	Baseline (month 0)	Follow-up (month 3)	Endpoint (month 6)	p-value
**Number of participants**				
Total (n)	175	101	100	-
Group 1: PA occupation and exercise (n, (%))	45 (25.7)	29 (28.7)	26 (26.0)	-
Group 2: PA occupation but non-exercise (n, (%))	48 (27.4)	30 (29.7)	34 (34.0)	-
Group 3: Non-PA occupation but exercise (n, (%))	38 (21.7)	17 (16.8)	19 (19.0)	-
Group 4: Non-PA occupation and non-exercise (n, (%))	44 (25.1)	25 (24.8)	21 (21.0)	-
**PA participation**				
**Meeting PA recommendations in PA occupation and exercise (Group 1)**	N=45	N=29	N=26	
Aerobic (n, (%))	45 (100.0)	22 (75.9)	21 (80.8)	**<0.001**[a]
Muscle-strengthening (n, (%))	31 (68.9)	7 (24.1)	16 (61.5)	**<0.001**[b]
Multicomponent (n, (%))	31 (68.9)	7 (24.1)	15 (57.7)	**<0.001**[b]
MVPA (median, IQR) [min/week]	225.0 (180.0, 360.0)	200.0 (150.0, 262.5)	205.0 (150.0, 236.2)	0.234[c]
**Meeting PA recommendations in PA occupation but non-exercise (Group 2)**	N=48	N=30	N=34	
Aerobic (n, (%))	0	9 (30.0)	17 (50.0)	**<0.001**[b]
Muscle-strengthening (n, (%))	5 (10.4)	6 (20.0)	16 (47.1)	**<0.001**[b]
Multicomponent (n, (%))	0	1 (3.3)	13 (38.2)	**<0.001**[a]
MVPA (median, IQR) [min/week]	45.0 (15.0, 90.0)	120.0 (0, 150.0)	150.0 (90.0, 180.0)	**<0.001**[c]
**Meeting PA recommendations in non-PA occupation but exercise (Group 3)**	N=38	N=17	N=19	
Aerobic (n, (%))	38 (100.0)	12 (70.6)	12 (63.2)	**<0.001**[a]
Muscle-strengthening (n, (%))	25 (65.8)	6 (35.3)	9 (47.4)	0.139[b]
Multicomponent (n, (%))	25 (65.8)	5 (29.4)	8 (42.1)	**0.048**[b]
MVPA (median, IQR) [min/week]	232.5 (160.0, 300.0)	200.0 (147.5, 262.5)	180.0 (135.0, 225.0)	0.086[c]
**Meeting PA recommendations in non-PA occupation and non-exercise (Group 4)**	N=44	N=25	N=21	
Aerobic (n, (%))	0	1 (4.0)	5 (23.8)	**<0.001**[a]
Muscle-strengthening (n, (%))	3 (6.8)	3 (12.0)	4 (19.0)	0.267[a]
Multicomponent (n, (%))	0	1 (4.0)	4 (19.0)	**0.004**[a]
MVPA (median, IQR) [min/week]	30.0 (0, 60.0)	60.0 (20.0, 90.0)	90.0 (60.0, 138.8)	**<0.001**[d]
**Body composition**				
**PA occupation and exercise (Group 1)**				
Body weight (median, (IQR)) [kg]	60.8 (54.6, 67.0)	61.9 (57.3, 65.7)	63.0 (56.1, 65.3)	0.891[c]
Body mass index (median, (IQR)) [kg/m^2]	23.7 (22.0, 26.9)	24.1 (22.4, 27.0)	24.0 (22.4, 26.0)	0.901[c]
Body fat percentage (median, (IQR)) [%]	32.2 (29.6, 36.1)	32.3 (29.7, 35.9)	30.6 (23.8, 34.7)	0.442[c]
Muscle mass percentage (median, (IQR)) [%]	32.2 (24.8, 38.5)	38.0 (35.6, 41.5)	39.3 (37.2, 47.4)	**<0.001**[c]
Visceral fat (median, (IQR)) [levels 1-9]	7.0 (5.5, 8.5)	7.0 (6.5, 9.5)	7.5 (6.5, 10.0)	0.428[c]
Resting metabolic rate (median, (IQR)) [kcal]	1266.0 (1130.0, 1370.0)	1195.5 (1113.5, 1345.0)	1227.0 (1154.0, 1431.0)	0.413[c]
**PA occupation but non-exercise (Group 2)**				
Body weight (median, (IQR)) [kg]	61.2 (55.7, 70.0)	62.7 (56.2, 70.0)	63.1 (57.1, 71.0)	0.762[c]
Body mass index (median, (IQR)) [kg/m^2]	24.8 (22.8, 28.2)	25.0 (22.9, 27.8)	25.7 (23.1, 29.0)	0.786[c]
Body fat percentage (median, (IQR)) [%]	35.0 (31.4, 38.9)	35.8 (31.6, 39.1)	36.6 (31.3, 41.3)	0.695[c]
Muscle mass percentage (median, (IQR)) [%]	32.2 (23.2, 37.8)	37.7 (36.1, 40.3)	37.7 (36.2, 42.6)	**<0.001**[c]
Visceral fat (median, (IQR)) [levels 1-9]	7.7 (6.4, 11.0)	7.5 (6.1, 9.9)	8.0 (6.5, 9.5)	0.914[c]
Resting metabolic rate (median, (IQR)) [kcal]	1246.0 (1145.8, 1387.5)	1170.0 (1129.0, 1305.2)	1224.0 (1135.0, 1331.0)	0.438[c]

**Non-PA occupation but exercise (Group 3)**				
Body weight (median, (IQR)) [kg]	63.4 (55.4, 68.8)	61.5 (49.1, 71.2)	65.8 (54.8, 75.2)	0.806[d]
Body mass index (median, (IQR)) [kg/m^2]	24.9 (22.3, 27.0)	25.6 (20.7, 27.5)	25.0 (22.9, 28.3)	0.859[c]
Body fat percentage (median, (IQR)) [%]	34.5 (31.2, 37.5)	31.5 (26.9, 39.1)	32.0 (26.1, 39.3)	0.854[c]
Muscle mass percentage (median, (IQR)) [%]	24.5 (23.1, 36.9)	37.4 (34.3, 40.9)	39.4 (36.0, 42.6)	**<0.001**[c]
Visceral fat (median, (IQR)) [levels 1-9]	7.5 (5.5, 9.9)	7.0 (4.5, 9.0)	8.0 (5.6, 11.0)	0.732[d]
Resting metabolic rate (median, (IQR)) [kcal]	1264.5 (1132.2, 1347.8)	1169.0 (1120.0, 1325.0)	1227.0 (1100.5, 1421.0)	0.608[d]
**Non-PA occupation and non-exercise (Group 4)**				
Body weight (median, (IQR)) [kg]	60.0 (54.9, 69.6)	58.6 (52.0, 60.6)	60.2 (55.0, 66.4)	0.287[c]
Body mass index (median, (IQR)) [kg/m^2]	23.8 (22.2, 27.9)	22.9 (21.3, 24.9)	24.2 (21.0, 26.8)	0.367[c]
Body fat percentage (median, (IQR)) [%]	33.6 (31.3, 37.3)	33.2 (30.2, 34.7)	32.4 (28.0, 33.6)	0.111[c]
Muscle mass percentage (median, (IQR)) [%]	25.4 (23.0, 35.6)	36.5 (33.1, 37.4)	36.6 (34.1, 39.9)	**<0.001**[c]
Visceral fat (median, (IQR)) [levels 1-9]	6.5 (5.4, 9.8)	6.5 (4.5, 7.0)	6.5 (5.8, 8.5)	0.467[c]
Resting metabolic rate (median, (IQR)) [kcal]	1223.0 (1109.8, 1336.0)	1143.0 (1019.0, 1201.0)	1168.0 (1079.0, 1222.5)	0.031[c]
**Physical fitness tests (passing scores)**				
**PA occupation and exercise (Group 1)**				
Flexibility (n, (%))	41 (91.1)	24 (82.8)	25 (96.2)	0.409[a]
Muscle strength and endurance (n, (%))	38 (84.4)	26 (89.7)	26 (100)	0.085[a]
Cardiovascular endurance (n, (%))	16 (35.6)	17 (58.6)	16 (61.5)	**0.040**[b]
**PA occupation but non-exercise (Group 2)**				
Flexibility (n, (%))	39 (84.7)	29 (96.7)	32 (94.1)	0.208[a]
Muscle strength and endurance (n, (%))	37 (78.7)	29 (96.7)	34 (100)	**<0.001**[a]
Cardiovascular endurance (n, (%))	18 (38.3)	25 (83.3)	16 (47.1)	**<0.001**[b]
**Non-PA occupation but exercise (Group 3)**				
Flexibility (n, (%))	35 (92.1)	17 (100)	17 (89.5)	0.591[a]
Muscle strength and endurance (n, (%))	35 (92.1)	15 (88.2)	19 (100)	0.656[a]
Cardiovascular endurance (n, (%))	28 (73.3)	13 (76.5)	12 (63.2)	0.478[b]
**Non-PA occupation and non-exercise (Group 4)**				
Flexibility (n, (%))	33 (75.0)	19 (76.0)	16 (76.2)	0.993[b]
Muscle strength and endurance (n, (%))	35 (79.5)	23 (92.0)	20 (95.2)	0.068[a]
Cardiovascular endurance (n, (%))	15 (34.1)	12 (48.0)	7 (33.3)	0.492[b]

[a]Fisher's exact test; [b]Chi-square test; [c]Kruskal-Wallis test; [d]ANOVA F-test. Bold p-values show statistical significance ($p<0.05$). IQR: interquartile range, METs: metabolic equivalents, MVPA: moderate- to vigorous-intensity physical activity, PA: physical activity.

Group 1: PA occupation and exercise (≥3.0 METs for most of the working time and ≥150 min/week of MVPA from exercise)

Group 2: PA occupation but non-exercise (≥3.0 METs for most of the working time and <150 min/week of MVPA from exercise)

Group 3: Non-PA occupation but exercise (<3.0 METs for most of the working time and ≥150 min/week of MVPA from exercise)

Group 4: Non-PA occupation and non-exercise (<3.0 METs for most of the working time and <150 min/week of MVPA from exercise)

Supplementary Table S2. Outcome measurements (last observation carried forward).

Outcome	Baseline (month 0)	Follow-up (month 3)	Endpoint (month 6)	p-value	Effect size (endpoint and baseline) (value, 95%CI)
**PA participation**					
**All groups (N=175)**					
Aerobic (proportion, (95%CI))	0.47 (0.40 to 0.55)	0.47 (0.40 to 0.54)	0.53 (0.46 to 0.60)	0.429[a]	1.12 (0.91 to 1.38)[w]
Muscle-strengthening (proportion, (95%CI))	0.36 (0.30 to 0.44)	0.28 (0.22 to 0.36)	0.40 (0.34 to 0.48)	0.057[a]	1.11 (0.85 to 1.44)[w]
Multicomponent (proportion, (95%CI))	0.32 (0.26 to 0.39)	0.21 (0.16 to 0.28)	0.37 (0.30 to 0.44)	**0.004[a]**	1.16 (0.87 to 1.55)[w]
MVPA (mean, (95%CI)) [min/week]	168.17 (135.25 to 201.09)	142.68 (120.80 to 164.57)	171.11 (142.72 to 199.51)	0.145[c]	-0.09 (-0.21 to 0.03)[y]
**PA occupation and exercise (Group 1) (N=45)**					
Aerobic (proportion, (95%CI))	1.00 (0.92 to 1.00)	0.84 (0.71 to 0.92)	0.89 (0.76 to 0.95)	**0.015[b]**	**0.89 (0.80 to 0.98)[w]**
Muscle-strengthening (proportion, (95%CI))	0.69 (0.54 to 0.80)	0.36 (0.23 to 0.50)	0.60 (0.45 to 0.73)	**0.004[a]**	0.87 (0.64 to 1.19)[w]
Multicomponent (proportion, (95%CI))	0.69 (0.54 to 0.80)	0.36 (0.23 to 0.50)	0.58 (0.43 to 0.71)	**0.005[a]**	0.84 (0.61 to 1.15)[w]
MVPA (mean, (95%CI)) [min/week]	349.00 (250.44 to 447.56)	229.33 (173.52 to 285.14)	282.89 (195.68 to 370.10)	0.217[c]	0.13 (-0.10 to 0.35)[y]
**PA occupation but non-exercise (Group 2) (N=48)**					
Aerobic (proportion, (95%CI))	0 (0 to 0.07)	0.21 (0.12 to 0.34)	0.35 (0.23 to 0.50)	**<0.001[a]**	**0.35 (0.22 to 0.49)[x]**
Muscle-strengthening (proportion, (95%CI))	0.10 (0.04 to 0.22)	0.17 (0.09 to 0.30)	0.33 (0.22 to 0.47)	**0.015[a]**	**3.2 (1.27 to 8.04)[w]**
Multicomponent (proportion, (95%CI))	0 (0 to 0.07)	0.02 (0 to 0.11)	0.27 (0.16 to 0.40)	**<0.001[b]**	**0.27 (0.14 to 0.40)[x]**
MVPA (mean, (95%CI)) [min/week]	50.00 (37.91 to 62.09)	69.38 (49.56 to 89.18)	124.79 (89.45 to 160.14)	**<0.001[c]**	**-0.48 (-0.65 to -0.26)[y]**
**Non-PA occupation but exercise (Group 3) (N=38)**					
Aerobic (proportion, (95%CI))	1.00 (0.91 to 1.00)	0.87 (0.73 to 0.94)	0.82 (0.66 to 0.91)	**0.021[b]**	**0.82 (0.70 to 0.95)[w]**
Muscle-strengthening (proportion, (95%CI))	0.66 (0.50 to 0.79)	0.53 (0.37 to 0.68)	0.60 (0.45 to 0.74)	0.500[a]	0.92 (0.65 to 1.30)[w]
Multicomponent (proportion, (95%CI))	0.66 (0.50 to 0.79)	0.50 (0.35 to 0.65)	0.89 (0.76 to 0.96)	0.378[a]	0.88 (0.62 to 12.6)[w]
MVPA (mean, (95%CI)) [min/week]	255.92 (221.62 to 290.22)	238.29 (196.42 to 280.16)	219.34 (179.92 to 258.77)	0.331[c]	0.20 (-0.06 to 0.44)[y]
**Non-PA occupation and non-exercise (Group 4) (N=44)**					
Aerobic (proportion, (95%CI))	0 (0 to 0.08)	0.02 (0 to 0.12)	0.11 (0.05 to 0.24)	**0.048[b]**	**0.11 (0.02 to 0.21)[x]**
Muscle-strengthening (proportion, (95%CI))	0.07 (0.02 to 0.18)	0.14 (0.06 to 0.27)	0.11 (0.05 to 0.24)	0.665[b]	1.67 (0.42 to 6.55)[w]
Multicomponent (proportion, (95%CI))	0 (0 to 0.08)	0.02 (0 to 0.12)	0.09 (0.04 to 0.21)	0.124[b]	0.09 (0 to 0.18)[x]
MVPA (mean, (95%CI)) [min/week]	36.36 (25.64 to 47.09)	51.47 (37.04 to 65.92)	65.68 (49.31 to 82.05)	**0.035[c]#**	**-0.64 (-1.08 to -0.21)[z]**
**Body composition**					
**All groups (N=175)**					
Body weight (mean, (95%CI)) [kg]	63.49 (61.66 to 65.32)	64.03 (62.12 to 65.93)	64.29 (62.32 to 66.25)	0.890[c]	-0.03 (-0.15 to 0.09)[y]
Body mass index (mean, (95%CI)) [kg/m^2]	25.30 (24.60, 26.00)	25.40 (24.70 to 26.11)	25.53 (24.80 to 26.26)	0.867[c]	-0.04 (-0.15 to 0.09)[y]
Body fat percentage (mean, (95%CI)) [%]	33.47 (32.40 to 34.54)	33.56 (32.35 to 34.77)	33.28 (32.03 to 34.54)	0.966[c]	0.01 (-0.11 to 0.13)[y]
Muscle mass percentage (mean, (95%CI)) [%]	30.94 (29.64 to 32.24)	34.67 (33.41 to 35.97)	35.17 (33.74 to 36.59)	**<0.001[c]**	**-0.25 (-0.36 to -0.13)[y]**
Visceral fat (mean, (95%CI)) [levels 1-9]	8.16 (7.52 to 8.79)	8.16 (7.59 to 8.73)	7.98 (7.26 to 8.70)	0.803[c]	0.02 (-0.10 to 0.13)[y]
Resting metabolic rate (mean, (95%CI)) [kcal]	1276.28 (1247.54 to 1305.03)	1257.79 (1228.89 to 1286.69)	1269.13 (1237.85 to 1300.41)	0.705[c]	0.04 (-0.08 to 0.15)[y]
**PA occupation and exercise (Group 1) (N=45)**					
Body weight (mean, (95%CI)) [kg]	63.04 (59.86 to 66.23)	63.47 (60.27 to 66.70)	63.42 (60.24 to 66.60)	0.971[c]	-0.02 (-0.25 to 0.21)[y]
Body mass index (mean, (95%CI)) [kg/m^2]	24.66 (23.47 to 25.84)	24.84 (23.61 to 26.07)	24.87 (23.69 to 26.05)	0.957[c]	-0.03 (-0.26 to 0.20)[y]
Body fat percentage (mean, (95%CI)) [%]	32.33 (30.24 to 34.43)	31.91 (29.27 to 34.55)	31.03 (28.63 to 33.43)	0.908[c]	0.04 (-0.19 to 0.28)[y]
Muscle mass percentage (mean, (95%CI)) [%]	32.60 (30.01 to 35.19)	36.93 (34.34 to 39.51)	37.52 (34.63 to 40.41)	**0.027[c]**	**-0.29 (-0.50 to -0.05)[y]**
Visceral fat (mean, (95%CI)) [levels 1-9]	7.73 (6.62 to 8.84)	7.92 (6.95 to 8.88)	7.90 (6.86 to 8.94)	0.812[c]	-0.07 (-0.30 to 0.19)[y]
Resting metabolic rate (mean, (95%CI)) [kcal]	1284.38 (1230.42 to 1338.33)	1256.29 (1205.13 to 1307.45)	1294.07 (1231.48 to 1356.65)	0.628[c]	-0.02 (-0.25 to 0.22)[y]
**PA occupation but non-exercise (Group 2) (N=48)**					
Body weight (mean, (95%CI)) [kg]	64.92 (61.20 to 68.65)	65.92 (61.92 to 69.92)	66.25 (62.29 to 70.22)	0.894[c]	-0.06 (-0.28 to 0.17)[y]
Body mass index (mean, (95%CI)) [kg/m^2]	26.05 (24.69 to 27.41)	26.19 (24.72 to 27.65)	26.46 (25.03 to 27.90)	0.895[c]	-0.05 (-0.28 to 0.18)[y]
Body fat percentage (mean, (95%CI)) [%]	34.20 (31.90 to 36.51)	34.84 (32.29 to 37.39)	35.21 (32.25 to 38.17)	0.739[c]	-0.10 (-0.32 to 0.14)[y]
Muscle mass percentage (mean, (95%CI)) [%]	31.16 (28.76 to 33.56)	34.98 (32.68 to 37.29)	35.68 (33.26 to 38.09)	**0.024[c]**	**-0.30 (-0.49 to -0.07)[y]**
Visceral fat (mean, (95%CI)) [levels 1-9]	9.03 (7.71 to 10.34)	8.67 (7.56 to 9.78)	8.23 (6.97 to 9.51)	0.915[c]	-0.10 (-0.32 to 0.14)[y]
Resting metabolic rate (mean, (95%CI)) [kcal]	1285.79 (1232.52 to 1339.06)	1270.60 (1217.92 to 1323.29)	1282.56 (1229.52 to 1335.60)	0.963[c]	0 (-0.18 to 0.27)[y]
**Non-PA occupation but exercise (Group 3) (N=38)**					
Body weight (mean, (95%CI)) [kg]	62.90 (58.86 to 66.95)	63.24 (59.01 to 67.48)	63.48 (58.98 to 67.99)	0.995[c]	-0.04 (-0.50 to 0.41)[z]
Body mass index (mean, (95%CI)) [kg/m^2]	25.43 (23.67 to 27.19)	25.37 (23.73 to 27.00)	25.34 (23.60 to 27.09)	0.999[c]	0 (-0.26 to 0.25)[y]
Body fat percentage (mean, (95%CI)) [%]	33.49 (30.88 to 36.10)	33.61 (30.95 to 36.28)	33.33 (30.63 to 36.02)	0.926[c]	0.05 (-0.20 to 0.30)[y]
Muscle mass percentage (mean, (95%CI)) [%]	30.30 (27.10 to 33.50)	33.37 (30.54 to 36.20)	33.89 (30.89 to 36.89)	0.167[c]	**-0.22 (-0.46 to -0.04)[y]**
Visceral fat (mean, (95%CI)) [levels 1-9]	7.61 (6.59 to 8.64)	7.54 (6.53 to 8.55)	6.97 (30.63 to 36.02)	0.673[d]	0.18 (-0.28 to 0.64)[z]
Resting metabolic rate (mean, (95%CI)) [kcal]	1266.05 (1206.29 to 1325.82)	1256.47 (1193.84 to 1319.10)	1249.26 (1185.32 to 1313.21)	0.927[d]	0.09 (-0.37 to 0.55)[z]

**Non-PA occupation and non-exercise (Group 4) (N=44)**					
Body weight (mean, (95%CI)) [kg]	62.90 (58.78 to 67.01)	63.21 (59.10 to 67.32)	63.72 (59.25 to 68.20)	0.875[c]	-0.02 (-0.26 to 0.21)[y]
Body mass index (mean, (95%CI)) [kg/m^2]	25.03 (23.53 to 26.53)	25.16 (23.68 to 26.64)	25.35 (23.74 to 26.96)	0.958[c]	-0.04 (-0.27 to 0.20)[y]
Body fat percentage (mean, (95%CI)) [%]	33.81 (32.04 to 35.58)	33.79 (31.81 to 35.76)	33.44 (31.56 to 35.32)	0.879[c]	0.04 (-0.20 to 0.28)[y]
Muscle mass percentage (mean, (95%CI)) [%]	29.56 (27.02 to 32.10)	33.15 (30.63 to 35.66)	33.31 (30.04 to 36.57)	0.096[c]	-0.22 (-0.44 to 0.02)[y]
Visceral fat (mean, (95%CI)) [levels 1-9]	8.11 (6.53 to 9.69)	8.37 (6.90 to 9.85)	8.65 (6.58 to 10.72)	0.850[c]	-0.03 (-0.26 to 0.20)[y]
Resting metabolic rate (mean, (95%CI)) [kcal]	1266.48 (1197.40 to 1335.56)	1246.48 (1175.40 to 1317.56)	1246.14 (1170.00 to 1322.27)	0.752[c]	0.10 (-0.14 to 0.32)[y]
**Physical fitness tests (passing scores)**					
**All groups (N=175)**					
Flexibility (proportion, (95%CI))	0.86 (0.80 to 0.90)	0.86 (0.80 to 0.90)	0.87 (0.82 to 0.92)	0.853[a]	1.02 (0.94 to 1.11)[w]
Muscle strength and endurance (proportion, (95%CI))	0.83 (0.77 to 0.88)	0.87 (0.81 to 0.91)	0.94 (0.89 to 0.96)	**0.010[a]**	**1.12 (1.04 to 1.21)[w]**
Cardiovascular endurance (%, (proportion %CI))	0.44 (0.37 to 0.52)	0.56 (0.48 to 0.63)	0.48 (0.41 to 0.56)	**<0.001[a]**	1.10 (0.88 to 1.38)[w]
**PA occupation and exercise (Group 1) (N=45)**					
Flexibility (proportion, (95%CI))	0.91 (0.79 to 0.96)	0.91 (0.79 to 0.96)	0.93 (0.82 to 0.98)	1.000[b]	1.02 (0.91 to 1.16)[w]
Muscle strength and endurance (proportion, (95%CI))	0.84 (0.71 to 0.92)	0.84 (0.71 to 0.92)	0.91 (0.79 to 0.96)	0.562[a]	1.08 (0.92 to 1.26)[w]
Cardiovascular endurance (%, (proportion %CI))	0.36 (0.23 to 0.50)	0.51 (0.37 to 0.65)	0.49 (0.35 to 0.63)	0.276[a]	1.38 (0.84 to 2.25)[w]
**PA occupation but non-exercise (Group 2) (N=48)**					
Flexibility (proportion, (95%CI))	0.85 (0.72 to 0.92)	0.88 (0.75 to 0.94)	0.92 (0.80 to 0.97)	0.584[a]	1.08 (0.93 to 1.26)[w]
Muscle strength and endurance (proportion, (95%CI))	0.79 (0.65 to 0.88)	0.88 (0.75 to 0.94)	0.96 (0.86 to 0.99)	**0.042[a]**	**1.22 (1.04 to 1.43)[w]**
Cardiovascular endurance (%, (proportion %CI))	0.38 (0.26 to 0.52)	0.67 (0.53 to 0.78)	0.48 (0.34 to 0.62)	**0.019[a]**	1.23 (0.78 to 2.00)[w]
**Non-PA occupation but exercise (Group 3) (N=38)**					
Flexibility (proportion, (95%CI))	0.92 (0.79 to 0.97)	0.92 (0.79 to 0.97)	0.89 (0.76 to 0.96)	1.000[b]	0.97 (0.84 to 1.12)[w]
Muscle strength and endurance (proportion, (95%CI))	0.92 (0.79 to 0.97)	0.92 (0.79 to 0.97)	0.97 (0.86 to 1.00)	0.696[a]	1.06 (0.95 to 1.18)[w]
Cardiovascular endurance (%, (proportion %CI))	0.74 (0.58 to 0.85)	0.74 (0.58 to 0.85)	0.66 (0.50 to 0.79)	0.681[a]	0.89 (0.66 to 1.20)[w]
**Non-PA occupation and non-exercise (Group 4) (N=44)**					
Flexibility (proportion, (95%CI))	0.75 (0.60 to 0.85)	0.73 (0.58 to 0.84)	0.75 (0.60 to 0.85)	0.961[a]	1.00 (0.78 to 1.27)[w]
Muscle strength and endurance (proportion, (95%CI))	0.80 (0.65 to 0.89)	0.84 (0.71 to 0.92)	0.91 (0.79 to 0.96)	0.326[a]	1.14 (0.96 to 1.36)[w]
Cardiovascular endurance (%, (proportion %CI))	0.34 (0.22 to 0.49)	0.34 (0.22 to 0.49)	0.34 (0.22 to 0.49)	1.000[a]	1.00 (0.56 to 1.79)[w]

P-values of [a]Chi-square test; [b]Fisher's exact test; [c]Kruskal-Wallis test; [d]ANOVA F-test. Effect sizes of [w]risk ratio; [x]risk difference; [y]Cliff's delta; [z]Cohen's d. Bold p-values show statistical significance ($p<0.05$). Bold effect sizes show statistical significance between endpoint and baseline.

95%CI: 95% confidence interval, METs: metabolic equivalents, MVPA: moderate- to vigorous-intensity physical activity, PA: physical activity.

Group 1: PA occupation and exercise (≥3.0 METs for most of the working time and ≥150 min/week of MVPA from exercise)

Group 2: PA occupation but non-exercise (≥3.0 METs for most of the working time and <150 min/week of MVPA from exercise)

Group 3: Non-PA occupation but exercise (<3.0 METs for most of the working time and ≥150 min/week of MVPA from exercise)

Group 4: Non-PA occupation and non-exercise (<3.0 METs for most of the working time and <150 min/week of MVPA from exercise)
